# Nanoparticle-mediated enhancement of plant cryopreservation: Cultivar-specific insights into morphogenesis and biochemical responses in *Lamprocapnos spectabilis* (L.) Fukuhara ’Gold Heart’ and ’Valentine’

**DOI:** 10.1371/journal.pone.0304586

**Published:** 2024-05-31

**Authors:** Dariusz Kulus, Alicja Tymoszuk, Alicja Kulpińska, Jacek Wojnarowicz, Urszula Szałaj

**Affiliations:** 1 Laboratory of Horticulture, Faculty of Agriculture and Biotechnology, Bydgoszcz University of Science and Technology, Bydgoszcz, Poland; 2 Laboratory of Nanostructures, Institute of High Pressure Physics, Polish Academy of Science, Warsaw, Poland; Center for Research and Technology Transfer, VIET NAM

## Abstract

The integration of nanoparticles (NPs) holds promising potential to bring substantial advancements to plant cryopreservation, a crucial technique in biodiversity conservation. To date, little attention has been focused on using nanoparticles in cryobiology research. This study aimed to assess the effectiveness of NPs in enhancing the efficiency of plant cryopreservation. In-vitro-derived shoot tips of bleeding heart (*Lamprocapnos spectabilis* (L.) Fukuhara) ‘Gold Heart’ and ‘Valentine’ were used as the plant material. The encapsulation-vitrification cryopreservation protocol included preculture, encapsulation, dehydration, storage in liquid nitrogen, rewarming, and recovery steps. Gold (AuNPs), silver (AgNPs), or zinc oxide (ZnONPs) nanoparticles were added at various concentrations either into the preculture medium or the protective bead matrix during encapsulation. The explant survival and further morphogenic and biochemical events were studied. Results showed that the impact of NPs on cryopreservation outcomes was cultivar-specific. In the ’Valentine’ cultivar, incorporating 5 ppm AgNPs within the alginate bead matrix significantly improved cryopreservation efficiency by up to 12%. On the other hand, the ’Gold Heart’ cultivar benefited from alginate supplementation with 5 ppm AgNPs and 5–15 ppm ZnONPs, leading to an over 28% increase in the survival rate of shoot tips. Interestingly, adding NPs to the preculture medium was less effective and sometimes counterproductive, despite promoting greater shoot proliferation and elongation in ‘Valentine’ explants compared to the control. Moreover, nanoparticles often induced oxidative stress (and enhanced the activity of APX, GPOX, and SOD enzymes), which in turn affected the biosynthesis of plant primary and secondary metabolites. It was found that supplementation of preculture medium with higher concentration (15 ppm) of gold, silver and zinc oxide nanoparticles stimulated the production of plant pigments, but in a cultivar-dependent matter. Our study confirmed the beneficial action of nanoparticles during cryopreservation of plant tissues.

## Introduction

Nanoparticles (NPs) have gained significant attention in the field of agriculture and horticulture due to their potential to revolutionize various aspects of farming and crop production. These tiny structures, typically measuring less than 100 nanometers, offer a range of applications [1]. They can be used to encapsulate and deliver essential nutrients and fertilizers directly to plant roots, ensuring more efficient nutrient uptake, reducing wastage and the need for excessive chemical application [2]. Nanoparticles can help improve water retention in soil, reducing the need for frequent irrigation, therefore, minimizing environmental impact [3]. Nanomaterials can also carry antimicrobial agents and biopesticides, offering a new approach to combat plant diseases and pests with increased precision [4]. Finally, nanosensors can monitor soil conditions, nutrient levels, and crop health in real-time, enabling farmers to make data-driven decisions for optimal crop management [5]. Nanoparticles are also widely used in plant biotechnology and *in vitro* cultures for the disinfection of explants, as growth stimulators and mutagen agents [6, 7].

The influence of nanoparticles on the *in vitro* development of plants has emerged as a significant area of research [8]. Nanoparticles may affect nutrient uptake, gene expression, and metabolome performance [9]. For instance, silver nanoparticles (AgNPs) have been shown to enhance seed germination rates and promote early seedling vigor in certain plant species [10, 11]. Similarly, titanium dioxide nanoparticles have demonstrated positive effects on root elongation and biomass accumulation during *in vitro* cultivation of soybean (*Glycine max* L.) [12]. Quantum dots, nanoscale semiconductor particles, have been employed to investigate and manipulate cellular processes, offering insights into the dynamics of plant cell division and differentiation [13]. Additionally, iron oxide nanoparticles have shown promise in facilitating the delivery of essential nutrients to plant cells during *in vitro* growth [14]. However, certain nanoparticles, such as zinc oxide nanoparticles (ZnONPs), may exhibit phytotoxic effects at elevated concentrations [15], underscoring the importance of careful dosage considerations in nanoparticle applications for optimal plant development.

Recent studies have demonstrated that certain metal and metal oxide nanoparticles can alter the physiological processes in plants, affecting photosynthesis, production of metabolites, and oxidative stress responses [16, 17]. For example, TiO_2_NPs have been shown to enhance the photosynthetic efficiency in *Raphanus sativus* L. plant [18], while AgNPs have exhibited both stimulatory and inhibitory effects on various biochemical pathways in different vegetable crops [10]). On the other hand, iron oxide nanoparticles (Fe_2_O_3_NPs) act as elicitors to stimulate the production of bioactive antioxidants and metabolites in the *in vitro* callus cultures of *Bergenia ciliata* (Haw.) Sternb [19]. These examples illustrate the diverse impact of nanoparticles on *in vitro* plant systems, emphasizing the need for understanding their precise effects to harness their full potential in agriculture and biotechnology. This is particularly important as only a few studies are comparing the effect of different types of nanoparticles (usually two) on the biochemical and physiological response of various plant cultivars [20].

Unlike micropropagation and breeding, the application of NPs in cryopreservation and biodiversity protection is scarce and reduced mostly to animal semen [21]. Plant cryopreservation, a vital technique in biodiversity conservation consisting of tissue storage in liquid nitrogen (LN), may gain significant advancements with the incorporation of nanoparticles. Nanoparticles due to their unique properties can improve the penetration and distribution of cryoprotectants within plant tissues [22], addressing the challenge of ice crystal formation and cellular damage during freezing and thawing. Their ability to scavenge reactive oxygen species and mitigate oxidative stress described by Zia-ur-Rehman et al. [23] may become invaluable in preventing cell damage and ensuring the recovery of cryopreserved plant materials. Moreover, ZnONPs, due to their high thermal conductivity may enhance the cooling and thawing rates, as observed with buffalo sperm samples, which has a great impact on the cells’ survivability [24]. Gold nanoparticles (AuNPs) have been employed to enhance the viability of plant cells and tissues post-cryopreservation [25]. Their effect, however, depended on the dose and time of application. It was observed that nanoparticles added at a low concentration (10 ppm) to the protective bead matrix enhanced the survival of the shoot tips in *Lamprocpnos spectabilis* ‘Valentine’. On the other hand, their addition into the recovery medium after thawing had a deleterious effect, especially if used at a high concentration (30 ppm). More studies in this regard are needed, including the use of other nanoparticle types and sizes, but at a concentration low enough to minimize their phyto- and genotoxic effects reported previously [6, 7, 9].

The aim of this study was to verify the effect of gold, silver and zinc oxide nanoparticles applied at various steps of the encapsulation-dehydration cryopreservation protocol on the recovery, morphogenesis and biochemical events in *Lamprocapnos spectabilis ‘*Gold Heart’ and ‘Valentine’. This innovative approach leverages the unique properties of nanoparticles to enhance the success and efficiency of cryopreservation methods.

## Materials and methods

### Characteristics of the tested nanoparticles

Materials: gold (III) chloride hydrate (Sigma-Aldrich, ≥49%), sodium borohydride (NaBH_4_, purity ≥ 96%, Sigma-Aldrich), sodium citrate C_6_H_5_Na_3_O_7_ 2H_2_O (Sigma-Aldrich, ≥99%), and tannic acid C_76_H_52_O_46_ (Fluka, Germany) were used as received. For an aqueous colloid preparation, deionized water obtained from the Deionizer Millipore Simplicity UV system was used (the specific resistivity of water was equal to 18.2 MΩ cm).

The AuNPs (6 nm particle size) were synthesized at the University of Lodz in water via the chemical reduction method. Briefly, chloroauric acid water solution (3.807 g, 0.136 wt. %) and water (25.179 g) were added to a flat bottom flask and mixed vigorously for 5 min at room temperature. Next, sodium borohydride (1.015 ml, 0.5 wt. %) was added, and the solution was mixed for an additional 1 h. Then 0.5 g of 10% sodium citrate and 0.195 g of 5% tannic acid were added. The final concentration of AuNPs in colloid was equal to 100 ppm.

The AgNPs (6 nm particle size) were prepared at the University of Lodz as follows: into 95.5 g of aqueous silver nitrate solution at the concentration of 0.017%, set on a mechanical stirrer, a mixture of sodium citrate (4.2 g, 4%) and tannic acid (0.63 g, 5%) was added. Immediately after mixing reagents, 0.7 g of solution of sodium borohydride, at the concentration of 2%, was added. After the addition of reductants, the color of the solution changed into brown. The whole mixture was vigorously stirred for 15 min. The final concentration of AgNPs in colloid was equal to 100 ppm.

The original microwave solvothermal synthesis procedure applied for the synthesis of ZnONPs was described in detail in our previous article [26]. The average particle sizes calculated on the basis of the specific surface area results was 25±2 nm.

### Culture medium and physical conditions in the growth room

The MS [27] medium, solidified with 0.8% (*w/v*) agar (Biocorp, Warsaw, Poland), was used in the experiments. The pH was adjusted to 5.8 (with 0.1 M HCl and 0.1 M NaOH) after adding all media components, before autoclaving at 105 kPa and 121°C for 20 min. The medium (40 mL) was poured into 350-mL glass jars sealed with plastic caps or 90-mm Petri dishes sealed with parafilm. Sucrose, NPs, and plant growth regulator (Sigma-Aldrich, St. Louis, MO, USA) concentration is provided in the specific stages of the experiment.

The cultures were kept in the growth room at 24°C ± 1°C, under 16-h photoperiod conditions and photosynthetic photon flux density of approximately 30.0 μmol m^-2^ s^-1^ provided by standard cool daylight TLD 54/36W fluorescent lamps (Koninklijke Philips Electronics N.V., Eindhoven, The Netherlands) with colour temperature of 6200 K, unless otherwise stated.

### Biological material and multiplication of plants

*In-vitro*-derived plantlets of bleeding heart (*Lamprocapnos spectabilis* (L.) Fukuhara) ‘Gold Heart’ and ‘Valentine’ were used as the source of explants. Axenic cultures of the two cultivars were obtained from the *in vitro* gene bank of the Laboratory of Horticluture, Faculty of Agriculture and Biotechnology, Bydgoszcz University of Science and Technology.

The donor plants, approximately 10–12 cm long, were cloned via the single-node method in the MS medium devoid of plant growth regulators, to produce the appropriate number of plant material. For this purpose, shoots were cut into nodal segments and subcultured on a fresh medium (seven explants per jar) every eight weeks for four months.

Shoot tips with 2–3 young leaves covering the meristem (1.0–2.0 mm in length) were used in the cryopreservation experiments.

### Cryopreservation procedure

The cryopreservation protocol included the following steps: preculture, encapsulation, dehydration, LN storage, rewarming, and recovery. Moreover, an additional *in vitro* rooting step was implemented (S1 Fig).

### Experiment I–effect of NPs added into the preculture medium

#### 1) Preculture

To produce the shoot tips, *in vitro*-derived single-node explants, with removed leaves, were cultured for one week on the solid MS medium with 9% (*w/v*) sucrose, 4.65 μM (1.0 mg L^-1^) kinetin (KIN) and 10 μM (2.62 mg L^-1^) of abscisic acid (ABA).

Silver, gold or zinc oxide nanoparticles, at 5 and 15 ppm, were poured onto the culture medium immediately after explants inoculation, 2 mL per jar. The control included explants non-treated with nanoparticles. Thus, seven experimental combinations were included.

Ten explants were inoculated into one jar. One culture jar was considered a single repetition. The experiment was repeated six times, i.e. a total of 840 shoot tips were used (420 for each cultivar; 60 per experimental combination).

#### 2) Encapsulation, dehydration, and LN storage

Shoot tips were excised with a micro-scalpel and binocular and embedded for 10 min in 3% (*w/v*) sodium-alginate based on the MS medium salts, without calcium II chloride (CaCl_2_), supplemented with 9% sucrose. Then, the beads, 3–4 mm in diameter, were hardened in 0.1 M CaCl_2_ solution for 30 min. The encapsulated explants were rinsed three times with distilled sterile water to remove the excess of CaCl_2_. Firm beads were osmoprotected with the loading solution (2.0 M glycerol and 0.4 M sucrose) for 20 min. Next, the explants were dehydrated with Plant Vitrification Solution 3 (PVS3; 50% glycerol and 50% sucrose, *w/v*) for 150 min at room temperature. Ten beads covered with PVS3 were placed in a 2.0 mL sterile cryovial, and directly immersed in LN.

#### 3) Rewarming, recovery, and rooting

After a day of storage, the cryovials were removed from LN and rewarmed rapidly in a water bath (39±1°C for 3 min). The PVS3 was removed from the vials with a pipette and the explants were rinsed with liquid MS medium with 1.2 M sucrose (for 30 min). Next, the still encapsulated shoot tips were inoculated on the MS recovery medium with 3% sucrose and 2.22 μM (0.5 mg L^-1^) 6-benzyladenine (BA) in a 90-mm Petri dish sealed with a parafilm. The cultures were kept in the growth room, in darkness. After 48 hours, the explants were transferred to a 16-h photoperiod and kept at the light intensity of approximately 13.0 μmol m^-2^ s^-1^ for 5 days (to prevent the phototoxic effect). Next, the shoot tips were transferred to the initial lighting conditions.

After 60 days of starting the recovery culture, apical shoot fragments with a few leaves were dissected and transferred to the rooting MS medium with 11.42 μM (2.0 mg L^-1^) indole-3-acetic acid (IAA) for 21 days.

### Experiment II–effect of NPs added into the protective bead matrix

Identical steps and parameters during the cryopreservation procedure were used in the second experiment as in the first one. The only difference was that there were no NPs added to the preculture medium. Instead, the AgNPs, AuNPs and ZnONPs, at 5 ppm and 15 ppm, were added into the sodium alginate solution (second step of the procedure; encapsulation). A control without NPs was also included in the second experiment.

### Evaluation of cryopreservation efficiency and biometrical analysis of plants in vitro

The share [%] of LN-derived explants regenerating shoots was evaluated after 30 days of recovery culture. The total number of dissected shoot tips was considered 100%. Moreover, the number and length of shoots, as well as the rooting effectiveness, root number and length of the longest root were measured after completing the *in vitro* phase of the experiments.

### Biochemical array: Determination of pigments in leaves

Spectrophotometric analysis of pigments from *ex-vitro*-grown plants was performed using fresh-leaf samples. Ten samples from each experimental treatment were prepared (a total of 260 samples, 130 per cultivar). The tissues were crushed in a porcelain mortar with the addition of a few milligrams of quartz sand. To extract anthocyanins, methanol containing 1% HCl (*v/v*) was used following the Arnon [28] method. Porphyrins, chlorophylls and carotenoids were extracted as described by Lichtenthaler [29] using 80% acetone (*v/v*). The obtained extracts were filtered through a funnel with filter paper into 10 mL volumetric tubes. The spectrophotometric analysis of extracts was performed in the NanoPhotometer NP80 (Implen GmbH, München, Germany).

Absorption maxima were defined for pigment-specific wavelengths (λ_max_): for anthocyanins at 520 nm, for carotenoids at 440 nm, and for chlorophyll *a* and *b* at 649 and 665 nm, respectively. The content of anthocyanins, chlorophylls, and carotenoids per gram of tissue fresh weight (FW) was calculated with the algebraic method following Arnon [28] and Lichtenthaler and Buschmann [30]. The concentration of protoporphyrin, Mg-protoporphyrin and protochlorophyllide was determined at 575, 590 and 628 nm according to Malik et al. [31].

### Biochemical array: Oxidative stress effects

#### a) Analysis of total phenolic content

Shoot samples were homogenized using a chilled mortar and pestle in methanol containing 1% HCl (*v/v*). Analysis of the total phenolic content was performed according to the Folin-Ciocalteau procedure [32] by mixing the phenolic extract with distilled water and Folin-Ciocalteau reagent. After incubation at room temperature, sodium carbonate solution and distilled water were added. The reaction mixture was incubated at 40°C for 30 minutes. Absorbance was measured at 765 nm wavelength. The total phenolic content was calculated using gallic acid as the calibration standard per gram of tissue fresh weight.

#### b) Determination of superoxide dismutase, ascorbate peroxide and guaiacol peroxidase activity

Shoot samples were homogenized in phosphate buffer (pH 7.4) containing 1 mM EDTA, 1 mM dithiothreitol (DTT), and 2% polyvinylpyrrolidone (PVP) according to Homaee and Ehsanpour [33]. The homogenates were centrifuged and supernatants were used to determine the activity of antioxidant enzymes and protein content.

Protein content was measured based on the Bradford method [34] with bovine serum albumin (BSA) as the standard.

Superoxide dismutase (SOD, EC 1.15.1.1) activity was determined as described by Giannopolitis and Ries [35] with modification by measuring its ability to inhibit the photochemical reduction of nitro blue tetrazolium chloride (NBT). The incubation mixture contained potassium phosphate buffer (pH 7.8), 0.015 mM riboflavin, 0.156 M methionine, 0.756 mM NBT, and the enzyme extract. The reaction was initiated by turning on the UV lamp and the absorbance was measured at a wavelength of 560 nm. The complete reaction mixture without the enzyme extract served as the control. SOD activity was expressed in units [U] of activity per 1 mg protein.

The guaiacol peroxidase (GPOX, EC 1.11.1.7) and ascorbate peroxidase (APX; E.C. 1.11.1.11) activities were measured spectrophotometrically according to Maehly and Chance [36] with modifications described by Nowogórska and Patykowski [37] and Nakano and Asada [38], respectively. The spectrophotometric analysis of extracts was performed at specific wavelengths: for GPOX at 470 nm and for APX at 290 nm. Enzymatic activity was expressed in units [U] of activity per 1 mg protein.

The entire biochemical analysis was performed in six replications for each experimental treatment (a total of 156 samples were analyzed). The spectrophotometric analysis was performed in the NanoPhotometer NP80.

### Statistical analysis

The experiments were set in a completely randomized design for two cultivars independently. Each of the two experiments (application of nanoparticles during the preculture /*prec*/ or encapsulation /*enc*/ step) included seven treatments; i.e. control, 5 ppm AgNPs, 15 ppm AgNPs, 5 ppm AuNPs, 15 ppm AuNPs, 5 ppm ZnONPs and 15 ppm ZnONPs,

The results were statistically analysed with one-way ANOVA, and the comparisons of means were made with Duncan’s Multiple Comparison Test (*P* ≤ 0.05) using Statistica 12.0 (StatSoft, Poland) and ANALWAR-5.2-FR tools.

## Results

### Effect of nanoparticles on explant survival and in vitro growth

Nanoparticles had a positive effect on the survival and recovery potential of LN-stored explants in *L*. *spectabilis*, although this effect was cultivar-specific (Table 1). Zinc oxide NPs (at both 5 and 15 ppm), as well as silver NPs (at 5 ppm), increased the recovery rate of ‘Gold Heart’ shoot tips by even 28.4% if added into the alginate bead matrix. On the other hand, supplementation of the preculture medium with nanoparticles usually negatively affected the survival of cryopreserved explants. None of the experimental treatments affected the proliferation of shoots (1.0–1.4 per explant), however, a tendency was observed suggesting that the addition of nanoparticles into the preculture medium (particularly at higher concentrations) stimulated the elongation of shoots (9.1–11.8 cm), whereas the presence of NPs in the alginate bead matrix reduced the shoot length (4,6–8.5 cm) (Table 1).

**Table 1:** Effect of silver (AgNPs), gold (AuNPs), and zinc oxide (ZnONPs) nanoparticles applied during the preculture (prec) or encapsulation (enc) step of the encapsulation-vitrification cryopreservation protocol on the recovery of shoot tips 30 days after rewarming, as well as the number and length of shoots (per explant) after 60 days of recovery culture in *Lamprocapnos spectabilis* ‘Gold Heart’ and ‘Valentine’.

	Recovery (%)	No. of shoots	Shoot length (cm)
**Treatment**	**Gold Heart**		
**control**	42.7 ± 4.66 cd	1.2 ± 0.06 a	8.9 ± 1.25 a-e
**5 ppm AgNPs prec**	24.2 ± 4.17 e	1.3 ± 0.09 a	10.0 ± 1.22 a-c
**15 ppm AgNPs prec**	27.6 ± 5.37 de	1.3 ± 0.12 a	10.3 ± 1.33 ab
**5 ppm AuNPs prec**	27.0 ± 5.11 e	1.1 ± 0.06 a	9.1 ± 1.43 a-e
**15 ppm AuNPs prec**	32.3 ± 5.61 c-e	1.1 ± 0.05 a	7.0 ± 0.57 b-f
**5 ppm ZnONPs prec**	25.3 ± 3.74 e	1.0 ± 0.04 a	9.3 ± 0.80 a-d
**15 ppm ZnONPs prec**	26.5 ± 5.22 e	1.2 ± 0.13 a	11.8 ± 2.07 a
**5 ppm AgNPs enc**	60.4 ± 4.85 a	1.1 ± 0.07 a	5.8 ± 0.69 d-f
**15 ppm AgNPs enc**	40.1 ± 4.37 c-e	1.0 ± 0.00 a	6.1 ± 0.50 c-f
**5 ppm AuNPs enc**	42.7 ± 3.62 cd	1.2 ± 0.09 a	5.3 ± 0.33 e-f
**15 ppm AuNPs enc**	45.3 ± 5.77 bc	1.1 ± 0.04 a	4.6 ± 0.25 f
**5 ppm ZnONPs enc**	57.5 ± 3.56 ab	1.4 ± 0.17 a	8.5 ± 1.63 a-f
**15 ppm ZnONPs enc**	71.1 ± 4.83 a	1.2 ± 0.11 a	6.8 ± 0.64 b-f
	**Valentine**		
**control**	50.2 ± 5.15 bc	1.2 ± 0.12 b	6.4 ± 0.84 c-e
**5 ppm AgNPs prec**	40.5 ± 4.55 c-e	1.2 ± 0.07 b	7.0 ± 0.55 cd
**15 ppm AgNPs prec**	34.9 ± 4.10 de	1.7 ± 0.16 a	10.1 ± 0.98 b
**5 ppm AuNPs prec**	40.0 ± 3.78 c-e	1.8 ± 0.08 a	13.2 ± 1.51 a
**15 ppm AuNPs prec**	22.5 ± 2.40 f	1.1 ± 0.07 b	6.4 ± 1.12 c-e
**5 ppm ZnONPs prec**	38.1 ± 3.19 c-e	1.1 ± 0.05 b	5.3 ± 0.91 c-f
**15 ppm ZnONPs prec**	29.2 ± 5.25 ef	1.2 ± 0.06 b	7.4 ± 0.81 c
**5 ppm AgNPs enc**	62.4 ± 5.65 a	1.2 ± 0.09 b	6.3 ± 0.96 c-f
**15 ppm AgNPs enc**	43.5 ± 4.19 cd	1.0 ± 0.00 b	3.7 ± 0.23 ef
**5 ppm AuNPs enc**	49.7 ± 2.58 bc	1.0 ± 0.00 b	3.9 ± 0.18 ef
**15 ppm AuNPs enc**	57.7 ± 3.88 ab	1.0 ± 0.03 b	3.6 ± 0.15 f
**5 ppm ZnONPs enc**	31.1 ± 2.33 d-f	1.1 ± 0.05 b	3.9 ± 0.25 ef
**15 ppm ZnONPs enc**	39.8 ± 2.78 c-e	1.2 ± 0.10 b	4.6 ± 0.69 d-f

* Each number represents the mean value ± standard error. Significant differences in values are determined by Duncan’s *post hoc* test (*P*<0.05). Values with at least one same letter are not statistically different.

As for bleeding heart ‘Valentine’, the highest recovery rates were found if 5 ppm AgNPs (62.4%) or 15 ppm AuNPs (57.7%) were added into the alginate bead matrix (Table 1). On the other hand, the supplementation of preculture medium with nanoparticles had a negative impact on the survival of shoot tips (22.5–38.1%), except for 5 ppm AgNPs and 5 ppm AuNPs treatments. The addition of 15 ppm AgNPs and 5 ppm AuNPs into the preculture medium stimulated the proliferation of shoots (1.7–1.8 per explant) in bleeding heart ‘Valentine’ compared to all other treatments (1.0–1.2). The application of nanoparticles affected also the elongation of shoots (Table 1). Overall, as in the ‘Gold Heart’ cultivar, and also in ‘Valentine, the presence of NPs in the preculture medium stimulated the elongation of shoots in contrast to alginate supplementation. Shoots produced with 15 ppm AuNPs in the preculture medium were over two-fold longer (13.2 cm) than the control (6.4 cm). Conversely, the addition of 15 ppm AuNPs into the alginate bead matrix resulted in the formation of the shortest shoots (3.6 cm).

Shoots from most experimental objects produced roots in the rooting medium with a 100% efficiency, except for the ‘Gold Heart’ cultivar treated with 15 ppm ZnONPs in the preculture medium, which encountered a 60% rooting efficiency (Table 2). The highest number of roots (9.63 per explant) and length of the longest root (28.80 mm) in bleeding heart ‘Gold Heart’ were found after adding 15 ppm AuNPs into the alginate bead or 15 ppm AgNPs into the preculture medium, respectively, however, no significant differences were found between the studied treatments. On the other hand, in bleeding heart ‘Valentine’, the addition of 15 ppm AgNPs into the preculture medium resulted in a lower number of roots produced (5.70) than in the control (12.20) but with no difference in the root length (Table 2).

**Table 2:** Effect of silver (AgNPs), gold (AuNPs), and zinc oxide (ZnONPs) nanoparticles applied during the preculture (prec) or encapsulation (enc) step of the encapsulation-vitrification cryopreservation protocol on the rooting efficiency, as well as the number of roots (per explant) and length of the longest root after 21 days in rooting medium in *Lamprocapnos spectabilis* ‘Gold Heart’ and ‘Valentine’.

	Rooting (%)	No. of roots	Root length (mm)
**Treatment**	**Gold Heart**		
**control**	100 ± 0.0 a	6.20 ± 1.55 a	18.20 ± 5.75 a
**5 ppm AgNPs prec**	100 ± 0.0 a	7.30 ± 1.33 a	20.30 ± 5.37 a
**15 ppm AgNPs prec**	100 ± 0.0 a	7.20 ± 0.73 a	28.80 ± 6.68 a
**5 ppm AuNPs prec**	100 ± 0.0 a	7.60 ± 1.36 a	24.10 ± 7.35 a
**15 ppm AuNPs prec**	100 ± 0.0 a	5.30 ± 0.82 a	21.30 ± 2.25 a
**5 ppm ZnONPs prec**	100 ± 0.0 a	7.70 ± 1.13 a	25.70 ± 5.85 a
**15 ppm ZnONPs prec**	60 ± 16.3 b	9.50 ± 1.90 a	22.50 ± 6.96 a
**5 ppm AgNPs enc**	100 ± 0.0 a	8.80 ± 0.73 a	22.70 ± 6.17 a
**15 ppm AgNPs enc**	100 ± 0.0 a	7.50 ± 1.09 a	22.60 ± 4.71 a
**5 ppm AuNPs enc**	100 ± 0.0 a	9.20 ± 1.15 a	20.50 ± 2.23 a
**15 ppm AuNPs enc**	100 ± 0.0 a	9.63 ± 1.16 a	19.13 ± 5.29 a
**5 ppm ZnONPs enc**	100 ± 0.0 a	7.20 ± 1.13 a	15.00 ± 2.55 a
**15 ppm ZnONPs enc**	100 ± 0.0 a	8.20 ± 0.76 a	19.10 ± 4.90 a
	**Valentine**		
**control**	100 ± 0.0 a	12.20 ± 1.69 a-d	35.90 ± 4.64 a
**5 ppm AgNPs prec**	100 ± 0.0 a	8.80 ± 1.36 c-e	29.10 ± 6.45 a
**15 ppm AgNPs prec**	100 ± 0.0 a	5.70 ± 1.26 e	18.70 ± 5.99 a
**5 ppm AuNPs prec**	100 ± 0.0 a	10.10 ± 1.85 b-e	15.90 ± 3.51 a
**15 ppm AuNPs prec**	100 ± 0.0 a	10.00 ± 1.17 b-e	32.80 ± 6.53 a
**5 ppm ZnONPs prec**	100 ± 0.0 a	8.00 ± 0.80 de	24.30 ± 3.89 a
**15 ppm ZnONPs prec**	100 ± 0.0 a	10.70 ± 1.97 b-d	27.70 ± 6.49 a
**5 ppm AgNPs enc**	100 ± 0.0 a	15.80 ± 1.31 a	27.90 ± 4.52 a
**15 ppm AgNPs enc**	100 ± 0.0 a	10.80 ± 1.21 b-d	25.60 ± 5.42 a
**5 ppm AuNPs enc**	100 ± 0.0 a	13.80 ± 1.11 ab	40.00 ± 6.00 a
**15 ppm AuNPs enc**	100 ± 0.0 a	15.40 ± 1.25 a	33.30 ± 4.79 a
**5 ppm ZnONPs enc**	100 ± 0.0 a	16.20 ± 1.93 a	26.60 ± 4.78 a
**15 ppm ZnONPs enc**	100 ± 0.0 a	13.10 ± 1.11 a-c	34.80 ± 13.99 a

* Each number represents the mean value ± standard error. Significant differences in values are determined by Duncan’s *post hoc* test (*P*<0.05). Values with at least one same letter are not statistically different.

### Effect of nanoparticles on the in vitro metabolic activity of LN-derived bleeding heart plants

**Fig 1:**
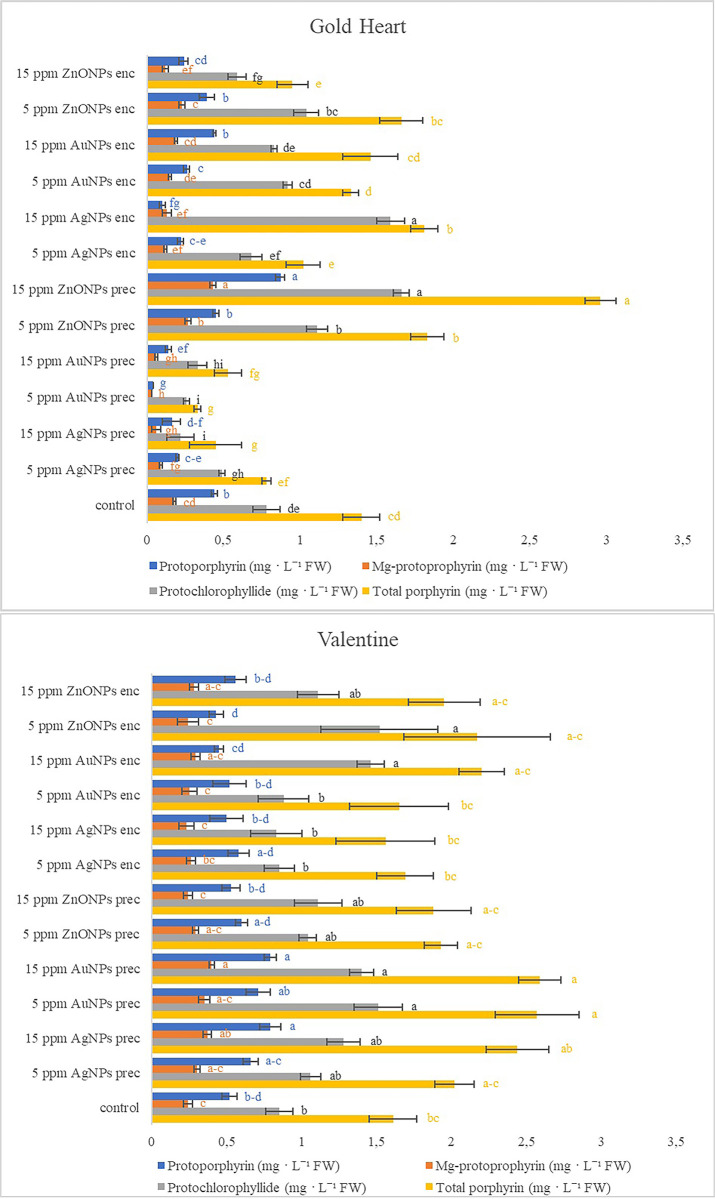
Effect of silver (AgNPs), gold (AuNPs), and zinc oxide (ZnONPs) nanoparticles applied during the preculture (prec) or encapsulation (enc) step of the encapsulation-vitrification cryopreservation protocol on the concentration of porphyrin forms in shoots after 60 days of recovery culture in *Lamprocapnos spectabilis* ‘Gold Heart’ and ‘Valentine’. Significant differences in mean values (± standard errors) are determined by Duncan’s *post hoc* test (*P*<0.05). Values with at least one same letter are not statistically different.

**Fig 2:**
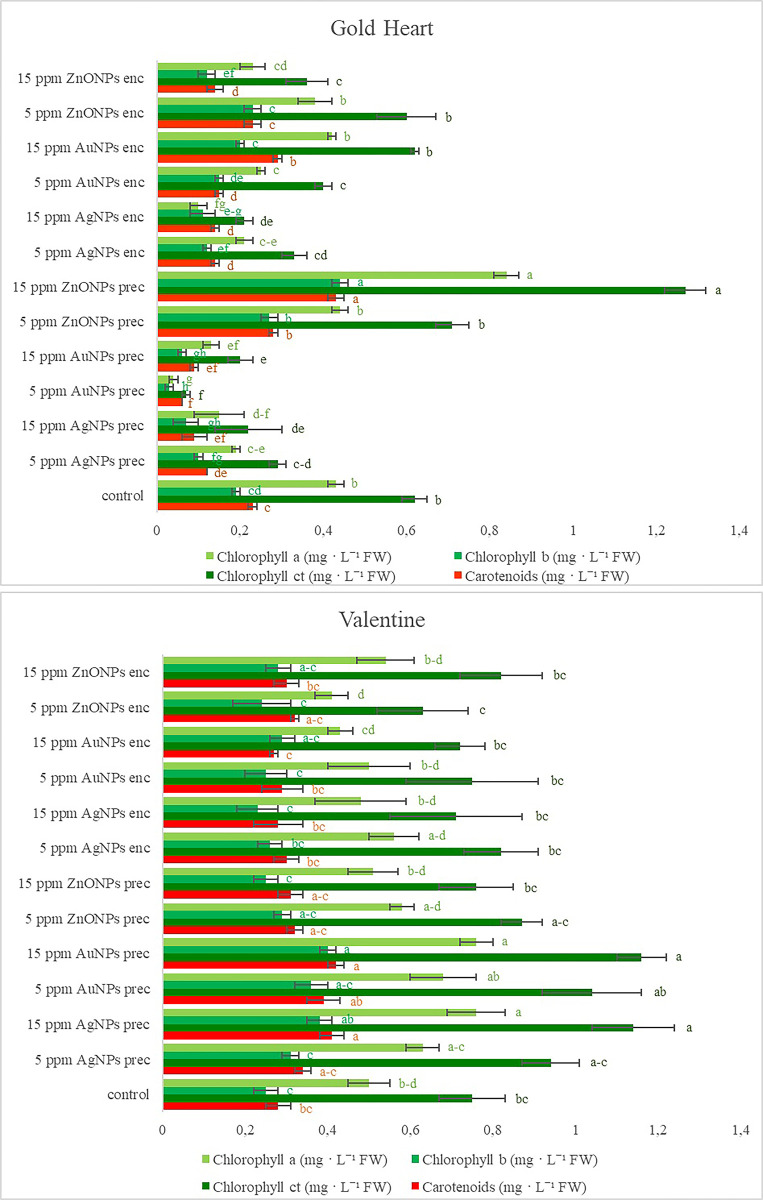
Effect of silver (AgNPs), gold (AuNPs), and zinc oxide (ZnONPs) nanoparticles applied during the preculture (prec) or encapsulation (enc) step of the encapsulation-vitrification cryopreservation protocol on the concentration of chlorophyll *a*, *b*, total (*ct*) and carotenoids in shoots after 60 days of recovery culture in *Lamprocapnos spectabilis* ‘Gold Heart’ and ‘Valentine’. Significant differences in mean values (± standard errors) are determined by Duncan’s *post hoc* test (*P*<0.05). Values with at least one same letter are not statistically different.

The metabolic profile of bleeding heart was affected by the nanoparticle treatment in a cultivar-dependent matter (Figs 1 and 2). The highest content of all studied porphyrin forms; i.e. protoporphyrin (0.87 mg g^-1^ FW), Mg-protoporphyrin (0.43 mg g^-1^ FW), protochlorophyllide (1.66 mg g^-1^ FW) and total porphyrin (2.96 mg g^-1^ FW); was found in the plants of bleeding heart ‘Gold Heart’ after adding 15 ppm ZnONPs into the preculture medium (Fig 1). The concentration of these pigments was approximately two-fold higher than in the control. Moreover, the addition of 5 ppm ZnONPs into the preculture medium stimulated the synthesis of Mg-protoporphyrin and protochlorophyllide, as well as increased the total porphyrin content. The concentration of protochlorophyllide and total porphyrins was also high after adding 15 ppm AgNPs into the alginate bead. On the other hand, several experimental treatments had a negative effect on the biosynthesis of porphyrins, especially protoporphyrin. The lowest contents of the studied porphyrin forms in bleeding heart ‘Gold Heart’ were found after adding 15 ppm AgNPs or 5/15 ppm AuNPs into the preculture medium (Fig 1).

Likewise, supplementation of preculture medium with 15 ppm of gold and silver nanoparticles stimulated the synthesis of protoporphyrin and Mg-protoprophyrin in bleeding heart ‘Valentine’ (Fig 1). On the other hand, a two-fold higher concentration of protochlorophyllide (1.40–1.52 mg g^-1^ FW), compared to the control (0.85 mg g^-1^ FW), was found in the experimental treatments: 5 and 15 ppm AuNPs in the preculture medium, as well as 15 ppm AuNPs and 5 ppm ZnONPs in the alginate beads. The highest total porphyrin content in ‘Valentine’ plants was reported after supplementing the preculture medium with 5 and 15 ppm AuNPs (2.57–2.59 mg g^-1^ FW *vs* 1.61 mg g^-1^ FW in the control). None of the NPs-treated plants contained significantly less porphyrin forms than the control in this cultivar (Fig 1).

The highest content of chlorophyll *a* (0.84 mg g^-1^ FW), chlorophyll *b* (0.44 mg g^-1^ FW) and total chlorophyll (1.27 mg g^-1^ FW) in cultivar ‘Gold Heart’ was found in the treatment 15 ppm ZnONPs in the preculture medium. These values were about two-fold higher than in the control. The addition of 5 ppm ZnONPs into the preculture medium additionally stimulated the biosynthesis of chlorophyll *b*. Some treatments had no effect on the synthesis of chlorophylls in this cultivar, but several others had a negative impact compared to the control (Fig 2). The lowest contents of chlorophylls in the ‘Gold Heart’ plants were found after supplementing the preculture medium with 5 or 15 ppm gold nanoparticles. Only in one experimental object (15 ppm AgNPs in the alginate bead), a significantly higher value of chlorophyll a/b ratio was found (5.95) than in the control (2.27) (Table 3).

**Table 3:** Effect of silver (AgNPs), gold (AuNPs), and zinc oxide (ZnONPs) nanoparticles applied during the preculture (prec) or encapsulation (enc) step of the encapsulation-vitrification cryopreservation protocol on the chlorophyll a/b ratio, chlorophyll to carotenoids ratio, as well as anthocyanins and total phenolics (TPC) content in shoots after 60 days of recovery culture in *Lamprocapnos spectabilis* ‘Gold Heart’ and ‘Valentine’.

	Chlorophyll a/b	Ct/carotenoids	Anthocyanins (mg g^-1^ FW)	TPC (mg g^-1^ FW)
**Treatment**	**Gold Heart**			
**control**	2.27 ± 0.05 b	2.65 ± 0.06 b	0.34 ± 0.04 de	4.23 ± 0.13 a
**5 ppm AgNPs prec**	1.96 ± 0.03 b	2.29 ± 0.05 d-f	0.44 ± 0.02 cd	8.59 ± 3.65 a
**15 ppm AgNPs prec**	2.58 ± 0.11 b	2.16 ± 0.08 f	0.43 ± 0.05 cd	5.19 ± 0.09 a
**5 ppm AuNPs prec**	1.47 ± 0.06 b	1.09 ± 0.05 g	0.44 ± 0.03 cd	5.28 ± 0.10 a
**15 ppm AuNPs prec**	2.12 ± 0.12 b	2.21 ± 0.07 ef	0.35 ± 0.03 de	4.45 ± 0.07 a
**5 ppm ZnONPs prec**	1.62 ± 0.01 b	2.54 ± 0.03 bc	0.81 ± 0.04 a	6.31 ± 0.29 a
**15 ppm ZnONPs prec**	1.91 ± 0.01 b	2.94 ± 0.01 a	0.49 ± 0.03 bc	4.93 ± 0.14 a
**5 ppm AgNPs enc**	1.81 ± 0.03 b	2.26 ± 0.06 d-f	0.31 ± 0.05 e	4.27 ± 0.17 a
**15 ppm AgNPs enc**	5.95 ± 2.94 a	0.90 ± 0.04 h	0.39 ± 0.03 c-e	6.05 ± 0.46 a
**5 ppm AuNPs enc**	1.72 ± 0.02 b	2.66 ± 0.03 b	0.38 ± 0.04 de	4.15 ± 0.27 a
**15 ppm AuNPs enc**	2.14 ± 0.02 b	2.40 ± 0.07 c-e	0.19 ± 0.04 f	4.68 ± 0.49 a
**5 ppm ZnONPs enc**	1.63 ± 0.05 b	2.62 ± 0.06 b	0.29 ± 0.02 e	5.19 ± 0.27 a
**15 ppm ZnONPs enc**	1.90 ± 0.06 b	2.42 ± 0.11 cd	0.56 ± 0.03 b	4.35 ± 0.20 a
	**Valentine**			
**control**	2.03 ± 0.05 b	2.65 ± 0.04 ab	0.90 ± 0.05 ab	5.55 ± 0.03 ab
**5 ppm AgNPs prec**	2.03 ± 0.01 b	2.74 ± 0.02 ab	0.62 ± 0.08 cd	5.21 ± 0.08 bc
**15 ppm AgNPs prec**	2.00 ± 0.02 b	2.74 ± 0.06 ab	0.52 ± 0.05 cd	5.30 ± 0.06 bc
**5 ppm AuNPs prec**	1.91 ± 0.01 b	2.66 ± 0.03 ab	0.57 ± 0.03 cd	4.87 ± 0.06 cd
**15 ppm AuNPs prec**	1.89 ± 0.02 b	2.78 ± 0.02 a	0.99 ± 0.06 a	5.33 ± 0.28 ab
**5 ppm ZnONPs prec**	1.97 ± 0.02 b	2.77 ± 0.03 a	0.64 ± 0.04 cd	4.57 ± 0.11 de
**15 ppm ZnONPs prec**	2.16 ± 0.10 b	2.40 ± 0.07 b	0.94 ± 0.07 ab	5.58 ± 0.30 bc
**5 ppm AgNPs enc**	2.10 ± 0.04 b	2.78 ± 0.03 a	0.74 ± 0.16 bc	5.39 ± 0.21 a-c
**15 ppm AgNPs enc**	2.05 ± 0.06 b	2.43 ± 0.08 ab	0.61 ± 0.04 cd	4.55 ± 0.08 de
**5 ppm AuNPs enc**	1.98 ± 0.03 b	2.51 ± 0.08 ab	0.99 ± 0.08 a	5.91 ± 0.17 a
**15 ppm AuNPs enc**	1.77 ± 0.32 b	2.65 ± 0.16 ab	0.46 ± 0.03 d	4.17 ± 0.17 ef
**5 ppm ZnONPs enc**	3.56 ± 0.95 a	1.94 ± 0.31 c	0.55 ± 0.09 cd	5.47 ± 0.23 ab
**15 ppm ZnONPs enc**	1.88 ± 0.02 b	2.66 ± 0.06 ab	0.47 ± 0.03 d	3.98 ± 0.22 f

* Each number represents the mean value ± standard error. Significant differences in values are determined by Duncan’s *post hoc* test (*P*<0.05). Values with at least one same letter are not statistically different.

As for the cultivar ‘Valentine’, the highest content of chlorophyll *a*, *b* and *ct* was found in the plants precultured on the medium with 15 ppm of gold or silver nanoparticles (Fig 2). None of the NP-treated plants contained less chlorophyll than the control. Only the plants from the experimental object, in which alginate was supplemented with 5 ppm ZnONPs, had an altered (increased) chlorophyll a/b ratio (3.56) compared to the control (2.03) (Table 3).

Most experimental treatments had a negative impact on the synthesis of carotenoids in bleeding heart ‘Gold Heart’ (0.06–0.15 mg g^-1^ FW) compared to the control (0.23 mg g^-1^ FW) (Fig 2). However, the addition of ZnONPs into the preculture medium, regardless of their concentration, and 15 ppm AuNPs into the alginate beads increased the content of these pigments in the plants (0.28–0.43 mg g^-1^ FW). Likewise, plants from most experimental treatments had a lower chlorophyll to carotenoids ratio (0.90–2.42) than the control (2.65) (Table 3). A significant increase in the value of this parameter (2.94) was found only after adding 15 ppm ZnONPs into the preculture medium. Application of ZnONPs either into the preculture medium (5 and 15 ppm) or alginate solution (15 ppm) enhanced the production of anthocyanins (0.49–0.81 mg g^-1^ FW) (Table 3). On the other hand, the content of these pigments was significantly lower in ‘Gold Heart’ plants (0.19 mg g^-1^ FW) if 15 ppm AuNPs were added into the alginate compared to the control (0.34 mg g^-1^ FW). No significant effect of nanoparticles on the total polyphenols content was found (Table 3).

Supplementation of preculture medium with 15 ppm of gold and silver nanoparticles stimulated the synthesis of carotenoids in *L*. *spectabilis* ‘Valentine’ (0.41–0.42 mg g^-1^ FW vs 0.28 mg g^-1^ in the control) (Fig 2). Only in one experimental treatment (15 ppm ZnONPs in the preculture medium), the chlorophyll to carotenoids ratio was decreased (1.94) compared to the control (2.65) in this cultivar (Table 3). None of the NPs-treated plants had a higher content of anthocyanins than the control (0.90 mg g^-1^ FW). Conversely, most ‘Valentine’ bleeding hearts had a decreased level of these pigments, with the lowest concentration (0.46 mg g^-1^ FW) reported after adding 15 ppm AuNPs or ZnONPs into the alginate capsule. Likewise, a higher concentration of nanoparticles (Ag, Au and ZnO) in the alginate bead decreased the total polyphenol content in LN-derived plants (Table 3).

### Effect of nanoparticles on the in vitro enzymatic activity of LN-derived bleeding heart plants

The application of nanoparticles affected significantly the activity of the three analyzed enzymes in both cultivars studied (Table 4). The highest activity of APX (7.5 U) in bleeding heart ‘Gold Heart’ was reported when 15 ppm AuNPs was added into the alginate bead. Moreover, supplementation of the preculture medium with a higher concentration (15 ppm) of nanoparticles (gold, silver and zinc) also increased the activity of this enzyme (5.2–5.8 U) compared to the control (2.1 U). Likewise, the addition of 5 and 15 ppm AgNPs into the preculture medium or 15 ppm AuNPs into the alginate bead matrix elevated the activity of GPOX (41.6–47.3 U) compared to the control (26.8 U). The activity of SOD was significantly higher in plants from the experimental treatments: 15 ppm AgNPs in the preculture medium (13.8 U) and 15 ppm AuNPs or 5/15 ppm ZnNPs in the alginate bead matrix (13.9–16.6 U), than in the control (10.9). There was a tendency suggesting that some NPs treatments could reduce the activity of the enzymes (even by 52%), although it was not confirmed statistically (Table 4).

**Table 4:** Effect of silver (AgNPs), gold (AuNPs), and zinc oxide (ZnONPs) nanoparticles applied during the preculture (prec) or encapsulation (enc) step of the encapsulation-vitrification cryopreservation protocol on the activity of ascorbate peroxidase (APX), glutathione peroxidase (GPOX), and superoxidase dismutase (SOD) in *Lamprocapnos spectabilis* ‘Gold Heart’ and ‘Valentine’ shoots after 12 weeks of recovery culture.

	APX (U)	GPOX (U)	SOD (U)
**Treatment**	**Gold Heart**		
**control**	2.1 ± 0.64 c-e	26.8 ± 3.15 de	10.9 ± 1.39 de
**5 ppm AgNPs prec**	4.5 ± 1.00 b-d	41.6 ± 6.09 a-c	9.4 ± 1.08 e
**15 ppm AgNPs prec**	5.8 ± 1.43 ab	42.7 ± 4.28 ab	13.8 ± 0.64 b
**5 ppm AuNPs prec**	2.2 ± 0.31 c-e	26.3 ± 1.71 de	9.8 ± 0.89 e
**15 ppm AuNPs prec**	5.6 ± 0.54 ab	27.2 ± 2.72 c-e	10.7 ± 1.60 e
**5 ppm ZnONPs prec**	5.1 ± 1.03 a-c	32.6 ± 3.66 b-e	12.1 ± 0.55 b-d
**15 ppm ZnONPs prec**	5.2 ± 1.20 ab	30.9 ± 4.04 b-e	13.6 ± 0.33 bc
**5 ppm AgNPs enc**	1.0 ± 0.24 e	25.6 ± 2.83 de	10.9 ± 1.16 de
**15 ppm AgNPs enc**	3.4 ± 1.00 b-e	30.5 ± 3.60 b-e	9.6 ± 0.51 e
**5 ppm AuNPs enc**	2.0 ± 0.26 de	23.8 ± 1.48 e	11.7 ± 0.75 b-d
**15 ppm AuNPs enc**	7.5 ± 1.79 a	47.3 ± 8.03 a	13.9 ± 0.15 b
**5 ppm ZnONPs enc**	3.1 ± 0.61 b-e	39.7 ± 7.64 a-d	16.6 ± 1.01 a
**15 ppm ZnONPs enc**	3.0 ± 0.28 b-e	26.4 ± 2.37 de	14.4 ± 0.29 ab
	**Valentine**		
**control**	0.5 ± 0.14 d	28.6 ± 4.37 b	11.0 ± 0.36 d
**5 ppm AgNPs prec**	1.8 ± 0.43 cd	40.5 ± 6.80 a	12.6 ± 0.72 b-d
**15 ppm AgNPs prec**	6.2 ± 1.98 a	25.6 ± 1.06 b	14.4 ± 1.01 ab
**5 ppm AuNPs prec**	2.0 ± 0.55 cd	19.2 ± 3.89 b	15.8 ± 1.08 a
**15 ppm AuNPs prec**	2.3 ± 0.34 cd	22.1 ± 2.29 b	14.3 ± 0.36 ab
**5 ppm ZnONPs prec**	3.9 ± 0.67 bc	26.1 ± 2.90 b	14.4 ± 0.20 ab
**15 ppm ZnONPs prec**	4.7 ± 1.02 ab	26.3 ± 7.33 b	15.5 ± 1.12 a
**5 ppm AgNPs enc**	0.6 ± 0.34 d	28.2 ± 5.72 b	11.8 ± 0.31 cd
**15 ppm AgNPs enc**	1.2 ± 0.34 d	17.3 ± 1.14 b	13.8 ± 0.57 a-c
**5 ppm AuNPs enc**	1.2 ± 0.35 d	16.6 ± 0.93 b	15.8 ± 0.92 a
**15 ppm AuNPs enc**	1.1 ± 0.24 d	18.3 ± 2.08 b	13.6 ± 0.82 a-c
**5 ppm ZnONPs enc**	1.0 ± 0.16 d	16.5 ± 1.29 b	13.5 ± 0.68 a-c
**15 ppm ZnONPs enc**	1.1 ± 0.34 d	24.7 ± 1.18 b	13.0 ± 0.87 b-d

* Each number represents the mean value ± standard error. Significant differences in values are determined by Duncan’s *post hoc* test (*P*<0.05). Values with at least one same letter are not statistically different.

As for the cultivar ‘Valentine’, the activity of antioxidant enzymes was much less diversified (Table 4). The lowest activity of APX was found in the control plants (0.5 U) and it was comparable to the treatments where nanoparticles were added into the alginate beads (0.6–1.2 U). Supplementation of preculture medium with NPs resulted in increased APX activity, with the highest value at 15 ppm AgNPs (6.2 U). On the other hand, increased GPOX activity was found only when 5 ppm AgNPs were added into the preculture medium. The lowest SOD activity was reported in the untreated control plants and most of the experimental treatments increased its activity, except for 5 ppm AgNPs in the preculture medium and 5 ppm AgNPs or 15 ZnONPs in the alginate matrix (Table 4).

## Discussion

### Effect of nanoparticles on explant survival and in vitro growth

Understanding the plant-nanoparticle interactions is crucial for harnessing the potential benefits of NPs in promoting plant growth and stress tolerance, while also addressing concerns about potential adverse effects. The results of the present study reveal the cultivar-specific impact of nanoparticles on the survival and morphogenesis of cryopreserved shoot tips in *Lamprocapnos spectabilis*.

The positive effect of nanoparticles, particularly ZnONPs at 5 and 15 ppm and AgNPs at 5 ppm, on the recovery rate of ’Gold Heart’ shoot tips when incorporated into the alginate bead matrix underscores the potential application of nanotechnology in cryopreservation protocols. Likewise, Kulus and Tymoszuk [25] demonstrated a positive impact of 10 ppm AuNPs on the survival of *L*. *spectabilis* ‘Valentine’ explants, when the nanoparticles were added to the alginate beads. This was explained by the high thermal conductivity of gold [39]. In the present study, augmentation of alginate with 5 ppm AgNPs increased the survival of bleeding heart ‘Valentine’ shoot tips, although AuNPs were less effective. The reason why gold nanoparticles were not as useful as in the study by Kulus and Tymoszuk [25] could be related to differences in the NPs concentration and size (6 *vs* 13 nm in diameter). As evident in the review by Wohlmuth et al. [40], the effect of nanoparticles is influenced by their size, shape and concentration. Moreover, the size of NPs affects their ability to be transported into plant tissue. Studies have confirmed that smaller NPs can enter the cell more easily and use more forms of transport [41], which could explain why smaller silver nanoparticles were more effective. Keeping in mind the importance of zinc as a micronutrient, it can be also elucidated why the addition of ZnONPs had a positive impact on the explants’ recovery *post-*LN-storage. Zinc contributes to cell proliferation and differentiation [42]. It was reported that ZnNPs greatly influence plant growth, yield, and fatty acid profiles of maize (*Zea mays* L.) [43]. According to Tymoszuk and Wojnarowicz [44], ZnONPs can be used as a growth regulator to stimulate *in vitro* germination and seedling development in onion (*Allium cepa* L.).

It is noteworthy that the supplementation of the preculture medium with nanoparticles generally exerted a negative influence on the survival of cryopreserved explants, in both cultivars studied, emphasizing the need for careful consideration of nanoparticle concentrations and the moment of their application. Cytotoxic effects of NPs in plants have been reported in numerous studies [45]. Exposure time is among the crucial factors that affect the nanoparticle-plant interaction [40]. Prolonged exposure to NPs in the preculture medium (for 7 days) before storage in LN could explain the obtained results.

Interestingly, a cultivar-dependent response was observed in the development of shoots, with a tendency indicating that in bleeding heart ‘Valentine’, nanoparticles, particularly 5 ppm AuNPs and 15 ppm AgNPs, stimulated shoot proliferation and elongation when present in the preculture medium, highlighting the potential for nanoparticle-mediated modulation of shoot development in this cultivar. Conversely, in both cultivars studied, NPs generally inhibited shoot elongation when incorporated into the alginate bead matrix. The observed dichotomy may be attributed to differences in the microenvironment as described by Tan et al. [46]. The microenvironment provided by the alginate bead matrix differs from the preculture medium in terms of physical structure, nutrient availability and water retention. These variations can affect NPs interactions with plant tissues and subsequent effects on shoot development. The release of nanoparticles from alginate is likely more sustained over time as reported by Fan et al. [47] in the case of amide nitrogen. This observation underscores the nuanced and complex interplay between nanoparticle concentration, exposure method, and plant response.

Moreover, the study demonstrates that the addition of nanoparticles can impact rooting efficiency in a cultivar-dependent manner, with ’Gold Heart’ cultivar showing reduced rooting efficiency when treated with 15 ppm ZnONPs in the preculture medium. On the other hand, despite the addition of 15 ppm AgNPs into the preculture medium stimulated shoot proliferation and elongation in bleeding heart ‘Valentine’, the same treatment resulted in a reduced number of roots produced. It can be, therefore, concluded that the application of nanoparticles influences specific signaling pathways differently in shoots and roots. Several factors may contribute to these divergent responses. For instance, NPs may influence the expression of genes related to shoot development but hinder the hormonal signals required for root initiation [48]. The stimulation of shoot elongation might be associated with enhanced activity of cytokinins, while inhibition of rooting could result from alternations in auxin biosynthesis [49]. Vinković et al. [50] revealed a significant increase in levels of *cis*-zeatin (ZEA) and isopentenyladenine (iP) type cytokinins in pepper (*Capsicum annuum* L.) exposed to AgNPs. On the other hand, similarly to our findings, Kumari et al. [51] reported that ZnONPs reduced the mitotic index and cell division in onion roots. Wang et al. [52] found that metal-based nanoparticles can cause damage to roots or be absorbed by plant roots, triggering oxidative stress, which in turn hampers root growth and nutrient uptake. The obtained results also correspond with the findings of Kulus and Miler [53], who described a cultivar-specific reaction of bleeding heart ‘Gold Heart’ and ‘White Gold’ shoot tips, both in terms of survival and morphogenesis, to cryopreservation protocols based on natural plant extracts.

### Effect of nanoparticles on the in vitro metabolic activity of LN-derived bleeding heart plants

The findings of this study highlight the cultivar-specific impact of nanoparticle treatments on the metabolic profile of *Lamprocapnos spectabilis*. Likewise, Tymoszuk [10] described that the biochemical parameters of developed seedlings in three vegetable species: *Solanum lycopersicum* L., *Raphanus sativus* L. var. *sativus* and *Brassica oleracea* var. *sabellica* varied within the same AgNPs treatments (including changes in the content of pigments and enzymatic activity). In bleeding heart ’Gold Heart,’ the significant increase in chlorophyll content, particularly with 15 ppm ZnONPs in the preculture medium, suggests a positive modulation of photosynthetic activity. This is positively surprising, as plants after LN storage often exhibit a lower chlorophyll content [54, 55]. Moreover, the increased concentration of porphyrin forms, carotenoids and anthocyanins indicates a potential role of zinc oxide nanoparticles in enhancing the biosynthesis of these essential pigments. Zinc is a crucial micronutrient that participates in various plant biological processes. The presence of Zn is essential for the synthesis of proteins, carbohydrates, and lipids, as well as for the metabolism of nucleic acids and antioxidants [56]. According to Hänsch et al. [57], its significance extends to chloroplast development and repair process of photosystem II when exposed to damage from light radiation, which could explain the obtained results. On the other hand, the negative impact of certain treatments, such as 15 ppm AgNPs or 5/15 ppm AuNPs in the preculture medium, on porphyrin levels in ‘Gold Heart’ underscores the need for precision in nanoparticle application strategies to avoid adverse metabolic effects. As for ‘Valentine’ cultivar, the positive influence of 15 ppm AgNPs and AuNPs in the preculture medium on the biosynthesis of porphyrins, chlorophylls and carotenoids could be attributed to the fact that metal nanoparticles might act as co-factors or catalysts in enzymatic reactions associated with pigment biosynthesis, such as phytoene synthase and lycopene cyclase, and activate signaling pathways related to photosynthesis [58]. Moreover, the increase in carotenoid content with certain nanoparticle treatments indicates a potential cultivar-specific role of nanoparticles in enhancing antioxidant defense mechanisms [59].

The observed alterations in pigment biosynthesis in *Lamprocapnos spectabilis* cultivars ’Gold Heart’ and ’Valentine’ in response to nanoparticle treatments coincide with changes in the activity of key antioxidant enzymes, namely ascorbate peroxidase (APX), guaiacol peroxidase (GPOX), and superoxide dismutase (SOD). These enzyme activities play crucial roles in cellular redox homeostasis and defense against oxidative stress, and their modulation can impact various metabolic pathways, including pigment biosynthesis [60].

In ’Gold Heart,’ the application of nanoparticles, particularly 15 ppm ZnONPs in the preculture medium, led to a significant increase in porphyrins and chlorophylls. This enhancement in pigment content correlated with elevated APX and GPOX activities, suggesting a potential link between antioxidant enzyme activity and the stimulation of porphyrin and chlorophyll biosynthesis [61]. The positive impact of ZnONPs on these pigments could be associated with the nanoparticles’ role in mitigating oxidative stress, thereby creating a favorable environment for porphyrin and chlorophyll production. Zinc functions as a cofactor for several enzymes, including RNA polymerase, superoxide dismutase, alcohol dehydrogenase, and carbonic anhydrase [62].

The significant increase in carotenoid content in the ‘Gold Heart’ cultivar in response to 15 ppm AuNPs in the alginate beads aligns with the enhanced activity of antioxidant enzymes. This relationship suggests that the nanoparticles could contribute to maintaining redox equilibrium, fostering an environment favorable to carotenoid biosynthesis [59]. These results could also explain why alginate supplementation with nanoparticles resulted in the highest recovery rates of LN-derived explants. The observed decrease in chlorophylls to carotenoids ratio, particularly after 15 ppm ZnONPs treatment, indicates a potential shift in the balance between these pigment classes influenced by nanoparticle-mediated oxidative stress [63].

In ’Valentine,’ the variations in APX, GPOX, and SOD activities in response to nanoparticle treatments were less diversified compared to ’Gold Heart.’ Nonetheless, the increased APX activity in the presence of 15 ppm AgNPs in the preculture medium may contribute to the observed higher chlorophyll content. The positive correlation between APX activity and chlorophyll biosynthesis suggests a potential protective role of this enzyme against oxidative damage, as suggested by Dhepe and Joshi [64], facilitating the efficient functioning of chloroplasts in bleeding heart. The obtained results also suggest that GPOX is the least, while SOD is the most sensitive marker of the redox status of cells of the two studied bleeding heart cultivars. A similar tendency was found in *Amaranthus tricolor* L. [65].

The obtained results underscore the importance of assessing the overall health and stress response of plants when utilizing nanoparticles in cryopreservation protocols. The varied response of APX, GPOX and SOD activities may indicate different regulatory mechanisms involved in the antioxidant defense system.

## Conclusions

The current study elucidates the impact of NPs on the survival, recovery potential, and biochemical activity of cryopreserved explants of *Lamprocapnos spectabilis*, ’Gold Heart’ and ’Valentine’. The results revealed a cultivar-specific response to nanoparticle treatments. Notably, zinc oxide and silver nanoparticles demonstrated a positive effect on the survival and recovery potential of shoot tip explants, particularly when incorporated into the alginate bead matrix. Bleeding heart ’Gold Heart’ exhibited increased recovery rates and shoot elongation, but with no effect on root formation in response to specific nanoparticle treatments, while ’Valentine’ displayed varying responses in terms of survival, shoot proliferation, and root development. Moreover, the biochemical analysis highlighted cultivar-dependent alterations in porphyrin, chlorophyll, carotenoid, anthocyanin, and polyphenol contents, highlighted by the altered redox status of the cells. These findings underscore the complexity of nanoparticle-plant interactions and call for further research to explore the underlying molecular mechanisms regulating these responses. Our study contributes valuable insights into the potential applications of nanoparticles in cryopreservation. Future research will focus on the *ex-vitro* performance of LN-derived plants treated with nanoparticles.

## Supporting information

S1 Fig Cryopreservation of *Lamprocapnos spectabilis* shoot tips. A–mother plants of bleeding heart ‘Valentine’; B–preculture of single node explants; C–preparation of alginate solutions with the addition of silver, gold and zinc oxide nanoparticles at various concentrations; D–dehydration of encapsulated explants in the PVS3 solution; E–encapsulated and dehydrated shoot tips in a cryovial; F–explants on the recovery medium post-LN-storage; G–LN-derived shoots of bleeding heart on the rooting medium.
(JPG)
