# Supplementary figures and images for: Nanoparticle-mediated enhancement of plant cryopreservation: Cultivar-specific insights into morphogenesis and biochemical responses in Lamprocapnos spectabilis (L.) Fukuhara ’Gold Heart’ and ’Valentine’
Source: PLoS One. 2024 May 31;19(5):e0304586. doi: 10.1371/journal.pone.0304586 (PMC11142695; doi:10.1371/journal.pone.0304586)

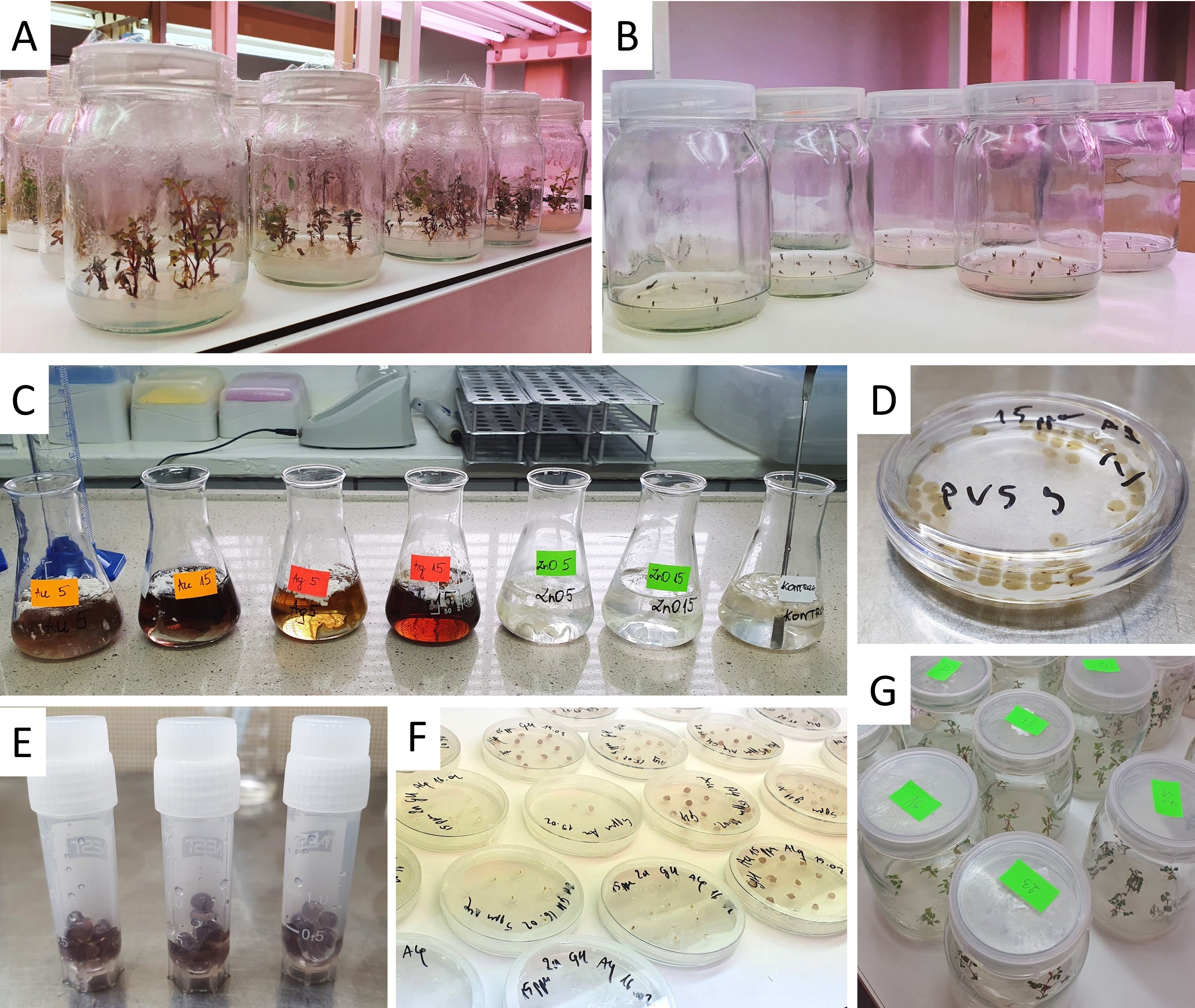

Supplement: S1 Fig — A–mother plants of bleeding heart ‘Valentine’; B–preculture of single node explants; C–preparation of alginate solutions with the addition of silver, gold and zinc oxide nanoparticles at various concentrations; D–dehydration of encapsulated explants in the PVS3 solution; E–encapsulated and dehydrated shoot tips in a cryovial; F–explants on the recovery medium post-LN-storage; G–LN-derived shoots of bleeding heart on the rooting medium. (JPG) [file pone.0304586.s001.jpg]
